# Bioelectrical understanding and engineering of cell biology

**DOI:** 10.1098/rsif.2020.0013

**Published:** 2020-05-20

**Authors:** Zoe Schofield, Gabriel N. Meloni, Peter Tran, Christian Zerfass, Giovanni Sena, Yoshikatsu Hayashi, Murray Grant, Sonia A. Contera, Shelley D. Minteer, Minsu Kim, Arthur Prindle, Paulo Rocha, Mustafa B. A. Djamgoz, Teuta Pilizota, Patrick R. Unwin, Munehiro Asally, Orkun S. Soyer

**Affiliations:** 1 Bio-Electrical Engineering Innovation Hub, University of Warwick, Coventry CV4 7AL, UK; 2 School of Life Sciences, University of Warwick, Coventry CV4 7AL, UK; 3 Department of Chemistry, University of Warwick, Coventry CV4 7AL, UK; 4 Department of Chemical and Biological Engineering, Northwestern University, Chicago, IL 60611, USA; 5 Department of Life Sciences, Imperial College London, South Kensington Campus, London SW7 2AZ, UK; 6 Department of Biomedical Engineering, School of Biological Sciences, University of Reading, Reading RG6 6AH, UK; 7 Clarendon Laboratory, Physics Department, University of Oxford, Parks Road, Oxford OX1 3PU, UK; 8 Department of Chemistry, University of Utah, 315 S 1400 E, Salt Lake City, Utah 84112, USA; 9 Department of Physics, Emory University, Atlanta, GA 30322, USA; 10 Centre for Biosensors, Bioelectronics and Biodevices (C3Bio), Department of Electronic and Electrical Engineering, University of Bath, Claverton Down, Bath BA2 7AY, UK; 11 Systems and Synthetic Biology Centre and School of Biological Sciences, University of Edinburgh, Alexander Crum Brown Road, Edinburgh EH9 3FF, UK

**Keywords:** bioelectricity, bioelectrical cell biology, cell physiology, cell biophysics, electrochemistry

## Abstract

The last five decades of molecular and systems biology research have provided unprecedented insights into the molecular and genetic basis of many cellular processes. Despite these insights, however, it is arguable that there is still only limited predictive understanding of cell behaviours. In particular, the basis of heterogeneity in single-cell behaviour and the initiation of many different metabolic, transcriptional or mechanical responses to environmental stimuli remain largely unexplained. To go beyond the *status quo*, the understanding of cell behaviours emerging from molecular genetics must be complemented with physical and physiological ones, focusing on the intracellular and extracellular conditions within and around cells. Here, we argue that such a combination of genetics, physics and physiology can be grounded on a bioelectrical conceptualization of cells. We motivate the reasoning behind such a proposal and describe examples where a bioelectrical view has been shown to, or can, provide predictive biological understanding. In addition, we discuss how this view opens up novel ways to control cell behaviours by electrical and electrochemical means, setting the stage for the emergence of bioelectrical engineering.

## 1. Introduction

Biological electrical phenomena were recognized by Luigi Galvani and his contemporaries in the eighteenth century through the study of animal muscles and the nervous system [1]. These early studies have led to the development of major fields, especially neuroscience and cardiology. Outside of these fields, however, studies of bioelectricity, i.e. electrical and electrochemical processes in cellular systems, have remained fragmented. While it was discussed as early as the 1970s that bioelectricity may be fundamental to understanding various cellular behaviours [2], the electrical investigations of cells were only focused on cellular bioenergetics [3,4]. The bioelectrical view of cells as a more general concept has remained confined to the fringes of biological research for five decades, during which molecular biology has made astonishing advancements in our understanding of cells and our ability to manipulate genes.

**Figure 1.**
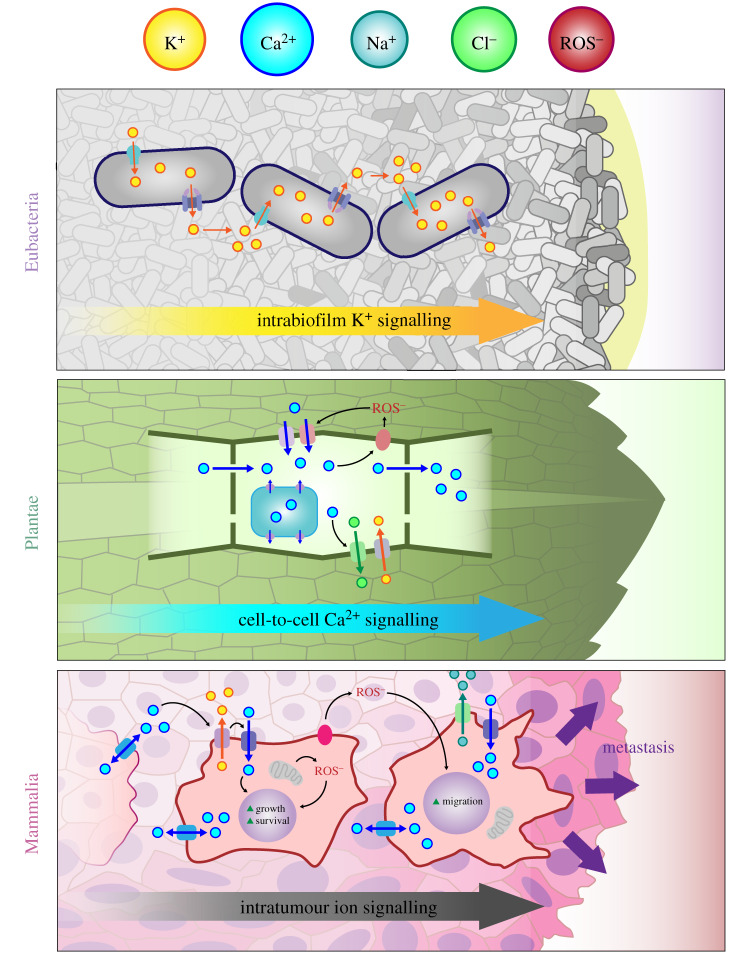
Recent research shows that both prokaryotes and eukaryotes use ion- and redox-based electrochemical signals for communication. It has been shown that such communication enables the organization of growth and developmental processes across multiple length scales.

Interestingly, the resulting findings from molecular studies highlight once again the importance of bioelectricity, and now there is a revival of a bioelectrical view of biological systems. Studies in diverse systems show that bioelectrical signals are at the heart of cell–cell communication in microorganisms, plants and animals (figure 1). Bioelectricity can underpin efficient growth and antibiotic resistance in bacterial biofilms [5,6] and organization, morphogenesis and regeneration in mammalian and plant tissues [7–9]. These findings, together with the realization that externally applied electrical fields can modulate multicellular processes such as regeneration in plant and vertebrate tissue [10–12], have resulted in the recent proposition that multicellular organization, and development more broadly, can, and should, be studied as a bioelectrical paradigm [13–15].

We argue here that bioelectricity can lead to a fundamental understanding of single-cell behaviours, beyond its roles in the multicellular context. By cell behaviours, we refer to high-level processes such as proliferation, dormancy and differentiation that are underpinned by dynamic changes in gene expression programmes, metabolic flux switching and mechanical cell properties. Notably, these changes are ultimately linked to the integrated physio-chemical properties of the electrochemically active cell–microenvironment interface. This motivates a bioelectrical view of the cell, the development of which can lead to a predictive understanding of cell behaviour.

## 2. Bioelectrical nature of the cell

**Figure 2.**
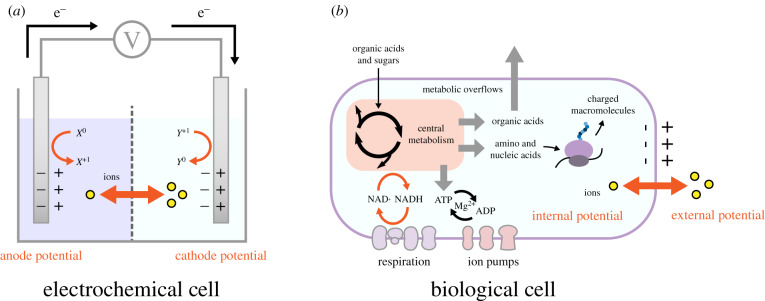
The basis for a bioelectrical view of cells can be motivated by drawing an analogy between a battery (*a*) and a biological cell (*b*). Both systems rely on ion flows and redox reactions across interfaces.

The bioelectrical conceptualization of cell behaviour can be illustrated with an analogy between a biological cell and a battery, both of which use redox reactions and ion movements (figure 2). In the case of the biological cell, its enclosed structure composed of multiple membranes partitions ions and charged and uncharged molecules (including macromolecules) across cellular compartments and within and outside the cell. The partitioning of charged molecules and ions gives rise to electrical and chemical potential differences across cellular membranes, and electrochemical gradients (ion motive forces, IMFs). A membrane potential (MP) arises from the combined electrical potential differences across a given membrane due to all charged molecules.

The maintenance of the MP and the coupling of IMFs to membrane-bound chemical reactions are key components of cellular bioenergetics and physiology, as recognized in the chemi-osmotic theory of respiration [3,4]. While the specific mechanisms of MP and IMF can be different across different membranes (e.g. mitochondrial versus plasma membrane in mammalian cells or the inner versus outer membranes in a bacterial cell [16]), their generation always involves electro-static and electro-dynamic processes [4]. IMFs can arise from selective transport or differential permeability to charged molecules, or can be generated through membrane-bound redox reactions of the respiratory chain [3,4]. A key point to recognize is that IMF and MP are coupled to each other and further to cell volume and metabolism [17,18]. The connection to volume arises from the distribution of ions and charged molecules (essential for the formation of MP), determining also the osmotic forces on cells [17]. The connection to metabolism is achieved by four general mechanisms (figure 2*b*). First, the steady-state concentrations of key metabolic redox and energy carrier pairs (NADH/NAD^+^ and ATP/ADP), which can determine metabolic pathway fluxes, can be altered by membrane-bound dehydrogenases and ATPases that can either use IMF to convert these pairs or use their conversions to sustain/generate it. Second, membrane transporters can couple the transport of metabolites, in particular organic acids and sugars, with the transport of ions, thereby linking this metabolically central process to IMF generation [2,19]. Third, several ‘master’ compounds within central metabolism—such as glutamate, which is involved in nitrogen assimilation and the synthesis of many other amino acids—can also act as gating molecules to control the state of ion channels, thereby influencing IMFs via MP [20]. Finally, the well-described excretions of metabolites and proteins from cells, as well as membrane-bound enzymatic processes, can influence the electrical and chemical potential of the cell microenvironment either directly or through redox reactions. In this context, it is worth noting that extracellular matrix polymers, such as collagen, chitin and cellulose, are shown to be piezoelectric [21].

## 3. Bioelectricity as a holistic approach to understand diverse cell behaviours

**Figure 3.**
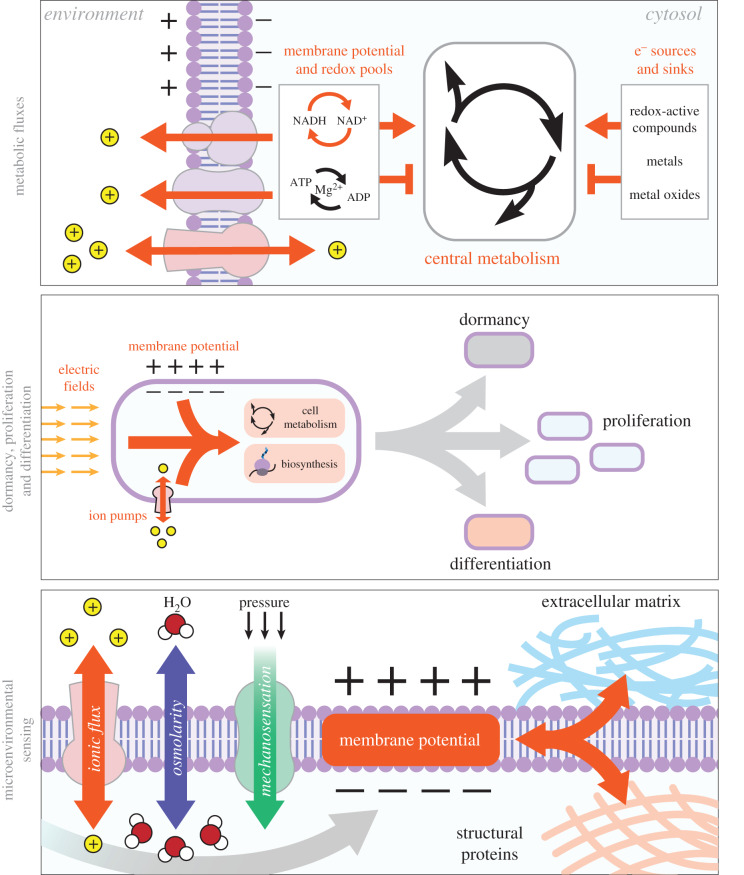
Cartoon illustration of the coupling between the bioelectrical nature of the cell, in particular MP and IMF, and higher level cellular behaviours.

The electrochemical nature of the cells and their microenvironments gives rise to a coupling between cell physiology and bioelectricity (i.e. MP and IMF) (figure 3). This bioelectrical conceptualization of the cell provides not only plausible explanations for many cell behaviours, but also a new framework to re-formulate much of the existing knowledge in cell physiology. To illustrate this point, we discuss here a few example cell behaviours in the bioelectrical context.

### 3.1. Metabolic fluxes

A key physiological response known as ‘the Warburg effect’ or ‘overflow metabolism’ is seen in many cell types including bacteria and is shown to underpin the behaviour of cancer cells and fermentative yeasts [22]. This effect involves the metabolic switching of cells from respiration to respiration and fermentation (i.e. respiro-fermentation), despite the presence of oxygen [22]. Respiration allows cells to generate mitochondrial IMFs through NADH oxidation/oxygen reduction and then harvest these for ATP synthesis [3,4]. This process releases more energy per carbon source than fermentation, which involves the reduction of NAD^+^. It is therefore puzzling that cells switch to respiro-fermentation in the presence of oxygen, when they could theoretically still respire and extract more energy per carbon. One explanation put forward to explain the Warburg effect is that it is a cellular strategy to maintain ‘optimal’ growth rates under limited enzyme capacity that has to be invested between biosynthesis, cell maintenance and fermentative/respiratory metabolism [23]. While this theory provides a plausible rationalization of the Warburg effect, protein allocation measurements across different growth conditions do not show major alterations to respiratory and fermentative enzyme allocations [24]. An alternative, simpler explanation can be proposed in the context of a bioelectrical framework: increasing respiration rates would result in an increased ATP/ADP ratio and a decreased NADH/NAD^+^ ratio, thereby reducing the thermodynamic feasibility of IMF generation and harvesting, which require NADH and ADP as substrates, respectively [3,4]. In other words, the respiration to respiro-fermentation switch could be underpinned by the thermodynamic feasibility of these two processes under a given MP value and based on the constrained NADH/NAD^+^ and ATP/ADP ratios. In line with this bioelectrical view, it has been indicated that cancer cells, known for their overflow metabolism, have altered MP levels [25] and that influencing the NADH/NAD^+^ ratio through engineered redox reactions directly alters the initiation point of the Warburg effect in bacteria and yeast [26,27]. While further work will ascertain the validity of this bioelectrical viewpoint to explain the Warburg effect, we note that this viewpoint is experimentally testable and offers a novel means to manipulate cellular metabolism through redox and MP manipulations.

Metabolism, more broadly, can be conceptualized as a bioelectrical process in its own right, as a coupled redox process in which electrons are transferred from an electron donor to an electron acceptor [18,28]. To facilitate this process, cells use a variety of electron sources and sinks, including redox-active compounds, metals and their oxides [29]. This opens up the possibility of using such compounds, or even electrode surfaces, to withdraw, or introduce, electrons into cellular metabolism [30]. While this possibility has already been realized in biotechnological research (e.g. cell-mediated bioelectrosynthesis or waste-to-electricity generation using microorganisms [29,30]), its true potential is in opening up new routes to study metabolism and its links to cell physiology through bioelectrical interfacing in mammalian and microbial cells. For example, redox-active, cell-permeable compounds have been used together with external electrodes to achieve an added, controllable redox cycling between cell metabolism and electrodes [31,32], thereby allowing the control of cellular physiological processes, such as the circadian clock [33] and gene expression [34].

### 3.2. Dormancy, proliferation and differentiation

The nature of the bioelectricity–metabolism coupling and its possible links to enabling different cell states are now being elucidated. In bacteria, it was recently shown that proliferating versus non-proliferating bacterial cells respond differently to electric fields [35]. In addition, it has been shown that MP responses are different when bacterial cell metabolism or biosynthesis is perturbed by carbon starvation or antibiotic treatment, respectively [36]. Other studies have also found that ionic fluxes can influence MP and physiological outputs such as the generation of metabolically dormant bacterial cells [37].

In mammalian cells, proliferating cancer cells are suggested to have altered plasma or mitochondrial MP (reviewed in [25]). Furthermore, MP is shown to be an indicator for stem-cell differentiation potential, alongside metabolic markers [38]. Hyperpolarization of the plasma MP is sufficient to induce differentiation [39], and, conversely, depolarization of MP in a differentiated cell can induce reversal to a multi-potent progenitor [40]. In this context, it is interesting that electrical fields can induce intracellular calcium oscillations in certain stem cells [41], possibly offering a route to control MP and differentiation in these cells.

### 3.3. Microenvironmental sensing

A coupling between bioelectricity and physiology is also evident in cellular sensing of internal and external conditions across all cell types. Osmoregulation of cell size can be achieved through both the production and consumption of key metabolites (e.g. glutamate) or through the alteration of ionic fluxes across the membrane [17]. Both processes can alter MP. For example, in bacteria, osmotic changes result in a significant motility response, possibly through changes in IMF causing flagellar rotation [42]. Such a coupling between flagellar rotation and IMF has been characterized [43] and recently been used to monitor changes in MP and cell metabolism through changes in flagellar rotation speeds [36].

There is a large family of voltage-gated ion channels and non-specific porins that can be influenced by MP and, in turn, can influence MP and IMF by their actions [44]. It is now increasingly clear that, in prokaryotic and eukaryotic cells, some ion channels can also respond to local mechanical forces within the membrane [45], thereby providing a direct link between MP, cell physiology and mechanical forces. The mechanical forces can come from the environment, including neighbouring cells within tissues or biofilms, or can be caused within the cell itself. Within the tissue context, for example, electrical and mechanical forces can act synergistically to re-model the extracellular matrix and further enable contractions of engineered muscle tissue [46]. Within cells, (de)stabilization of microtubules is implicated to alter the mitochondrial MP in cancer cells [47], while, in bacteria, the alteration of MP can change the membrane distribution of structural proteins involved in cell division [48,49]. It has also been suggested that action potentials in neurons are accompanied by a mechanical wave (soliton wave) across the membrane [50]. These examples highlight the possibility that the link between MP and mechanical forces can provide a key integrator of information from external and internal sources. Interestingly, one such process that potentially requires the integration of internal and external clues—the initiation of bacterial sporulation—was linked to bioelectrical changes in a recent study [51]. Whether such bioelectrical changes during sporulation are caused by mechanical forces and/or involve other contributing factors remains an open research question to pursue.

In plants, the best characterized electrical signalling responses are to mechanical stimuli caused by wounding and herbivory [9]. A landmark study demonstrated that electrical potentials were rapidly initiated upon wounding, which propagated to systemic leaves, initiating the classical jasmonate-based wound response [52]. Such wound-induced ‘electrical’ signals in plants are known as variation potentials (VPs) and show slower dynamics than action potentials seen in neurons. VPs appear to facilitate information transfer on a large scale, alerting all the intervening tissue of distal stimuli [53], and arise from the activation of ligand-dependent or mechano-sensitive calcium channels, facilitating Ca^2+^ influx. These wound VPs were shown to be dependent upon glutamate-like receptors (GLRs), which facilitate the generation of waves of Ca^2+^. Consistent with this finding, glutamate triggers long-distance, Ca^2+^-mediated plant defences against herbivory via GLRs [54]. Notably, the application of a synthetic electrical stimulus of similar magnitude and duration to wound VPs activated transcriptional re-programming remarkably similar to that activated systemically following wounding. This study thus provides tantalizing evidence for using bioelectrical stimuli to engineer beneficial responses in plants.

## 4. Bioelectrical engineering of cell biology: potential and challenges

The above-highlighted couplings between cell physiology and bioelectricity can only be pursued experimentally through the development of integrated quantitative measurement techniques. To this end, electrochemistry offers a range of quantitative techniques that are capable of measuring the concentration of ions (e.g. H^+^ (i.e. pH), Ca^2+^ or Na^+^) or specific redox-active compounds (e.g. neurotransmitters, glucose, flavins, hydrogen peroxide and oxygen). The use and combination of such techniques have already resulted in the development of specific and powerful bio-electrochemical tools. Multi-electrode arrays allow the measurement of electrochemical events occurring at time scales of the order of microseconds [55] and at the tissue level [56], while scanning ion conductance (SICM) and scanning electrochemical microscopy (SECM) allow mapping of ionic conductivity and redox reactions, respectively, at nano-scale to single-cell levels [57].

Electrochemical approaches can also be used to modify the chemical composition of the cell microenvironment in a selective and controlled manner by producing or delivering reactant species that will trigger specific processes [58,59] or by exposing cells to electrical fields and pulses [60–62]. This opens up the possibility of ‘dialling in’ on cellular behaviour at the single-cell and tissue levels. It has been shown for example that electric fields can influence or stop mammalian cell division [61,63] or trigger specific cellular responses [60,64] and can be used to distinguish between metabolically active and dormant bacterial cells [35]. Techniques such as SECM can directly deliver redox-active compounds to individual cells and measure their responses; for example, to interrogate the metabolism of cancerous versus normal cells [65].

Regardless of the specifics of a given technique, using electrochemical approaches to study and engineer cell behaviour poses challenges. By its nature, an electrochemical measurement is complex, involving processes at the interface of an electrode and solution. In the case of cellular methods, there would even be multiple interfaces among cells, solution and electrodes. Thus, the characteristics of such interfaces must be considered to correctly evaluate the measurements. At single- and sub-cell scales, untangling the interface effects can be complicated and requires the application of modelling approaches, such as finite-element modelling to solve coupled mass transport–reactivity problems [57,66]. The application of measurement and modelling in tandem provides a roadmap for elucidating cell/solution interface properties, as has been shown for understanding surface charge heterogeneities at the sub-cellular level [66]. Another challenge for the application of electrochemical techniques is signal acquisition and processing. Recent efforts have focused on improving signal sensitivity (both signal/noise ratio and the specificity of signal) and temporal resolution. For example, it is possible to minimize electrode impedance [67] by optimizing electrode size, morphology and materials. The use of such low-noise measuring systems revealed minute, yet regular, membrane capacitive current oscillations across large populations of mammalian cells, which have previously been considered electrically quiescent, such as C6 glioma cells [67,68].

A number of technical challenges need to be considered when combining electrode measurements with cells and tissues, including sample preparation, interfaces of electrodes for *in vitro* or *in vivo* studies and maintenance of the integrity of the sensing/stimulating electrodes and the living system. For example, for *in vitro* spatially resolved electrochemical measurements, cells must be immobilized, which may not reflect their natural state. Irrespective of the application (sensing or modifying cell behaviour), the electrode should not be cytotoxic to cells. It must be ensured that the physiological medium used neither is prohibitive for specific electrochemical techniques nor interferes with the measurement. By-products of a measurement (e.g. in the case of redox-based measurements) should not drastically disturb the cell microenvironment. This can be of particular concern, as by-products and coupled reactions, such as solvent breakdown, could impact and obscure experimental conclusions.

These challenges are far outweighed by the potential of developing a bioelectrical basis of cell behaviour. As we argued above, this will not only open up completely new ways of understanding cells, but also allow the development of novel bioelectrical means to control and engineer cell behaviour. The latter is happening already with emerging applications of so-called electroceuticals to treat biofilms and cancer tumours [61,69–71]. On the former premise, the ability to use nano- and micro-scale electrodes allows the elucidation of single-cell properties and responses in ways previously not possible. For example, recent applications of SICM have revealed cellular charge heterogeneities [66], which cannot be detected with bulk methods such as zeta-potential measurements of cells [72]. SECM combined with SICM opens up new methods for stimulating cells with ions or redox-active compounds and following their bioelectrical responses in real time and space [73]. The combination of such single-cell electrochemical measurements with other existing methods, such as fluorescence microscopy of ion-binding or MP-responsive dyes [6,35,74,75], and the tandem use of these measurements with modelling will provide a significantly improved theoretical basis for understanding the cell–ionic environment interface. The continued developments taking place in electrode manufacturing, electrochemical techniques and signal processing will enable bioelectrical control of cells and their behaviour at single-cell, tissue and perhaps even organ levels, using also implantable devices. These exciting prospects will require a true integration of cell biology, electrochemistry, physics and engineering in the years to come.

## 5. Concluding remarks

A bioelectrical view on cell behaviour, as advocated here, can help to establish a unifying framework that allows us to see cell behaviour arising from interfaces with its microenvironment through the fluxes of ions and redox-active compounds. This framework offers a new synthesis that can bridge molecular studies with a bioelectrical basis of cellular physiology. The resulting science can have a transformative effect on our understanding of cellular behaviour and pave the way to its direct control through predictive bioelectrical engineering, a process that is already advancing in the context of neuronal systems [76].

## Data accessibility

This paper has no additional data.
